# Prevalence and Determinants of Immediate and Long-Term PTSD Consequences of Coronavirus-Related (CoV-1 and CoV-2) Pandemics among Healthcare Professionals: A Systematic Review and Meta-Analysis

**DOI:** 10.3390/ijerph18042182

**Published:** 2021-02-23

**Authors:** Buthaina al Falasi, Mouza al Mazrouei, Mai al Ali, Maithah al Dhamani, Aisha al Ali, Mariam al Kindi, Murat Dalkilinc, Mai al Qubaisi, Luciana Aparecida Campos, Hashel al Tunaiji, Ovidiu Constantin Baltatu

**Affiliations:** 1 Zayed Military Hospital, Abu Dhabi P.O. Box 3740, United Arab Emirates; buthaina.alfalasi@gmail.com (B.a.F.); mouza.dr@gmail.com (M.a.M.); Mai.alali88@hotmail.com (M.a.A.); maithah.aldahmani@gmail.com (M.a.D.a.); maialali8891@gmail.com (A.a.A.); mariam.obaid.alkindi@gmail.com (M.a.K.); ptmurat@hotmail.com (M.D.); la2albi@hotmail.co.uk (M.a.Q.); 2 College of Health Sciences, Abu Dhabi University, P.O. Box 59911, United Arab Emirates; luciana.camposbalta@adu.ac.ae; 3 Center of Innovation, Technology and Education (CITE) at Sao Jose dos Campos Technology Park, Sao Jose dos Campos 12247-016, Brazil; 4 Institute of Biomedical Engineering, Anhembi Morumbi University-Laureate International Universities, Sao Jose dos Campos 12247-016, Brazil; 5 College of Medicine and Health Sciences, Khalifa University, Abu Dhabi P.O. Box 127788, United Arab Emirates

**Keywords:** coronavirus, stress disorders, immediate and long-term PTSD, health personnel, risk factors, odds ratio

## Abstract

Background: The COVID-19 pandemic continues to rise. In order to control the COVID-19 pandemic, healthcare professionals have been subjected to increased exposure to work stress. In this systematic review, we aimed at investigating the prevalence and determinants of immediate and long-term post-traumatic stress disorder (PTSD) effects on healthcare professionals by the COVID-19 (SARS CoV-2) and SARS-2003 (SARS CoV-1) pandemics. Methods: This systematic review was conducted according to the recommendations of the Protocols for Systemic Review and Meta-Analysis (PRISMA) statement. Only studies reporting the prevalence of PTSD (frequency, percentage) and related risk factors (adjusted odds ratio (OR)) in healthcare professionals (HCPs) during the SARS CoV-2 and SARS CoV-1 pandemics were included. The following databases were screened: Medline, Embase, PsychINFO, and Health Psychosocial Instrument (HaPI). Results: Six of eight studies reported PTSD symptoms among healthcare professionals during the COVID-19 pandemic in China (three), Singapore (one), India (one), and the United States of America (USA) (two), while two studies reported symptoms during the SARS-2003 pandemic in China (one) and Singapore (one). Sample sizes ranged from 263 to 5062 with a combined total of 10,074 participants. All of the studies self-reported the level of exposure to coronaviruses (CoV-1 and CoV-2) and severity of PTSD. Seven studies reported the prevalence of immediate PTSD and determinants, while one study reported delayed-onset PTSD (3 years after CoV-1 pandemic). Determinants of immediate PTSD were reported for the CoV-2 pandemic, while those for long-term PTSD were reported for the CoV-1 pandemic. Conclusions: A comprehensive understanding of the prevalence and determinants of immediate or long-term pandemic PTSD for healthcare workers can improve prevention, diagnosis, and management. Rigorous research measuring the prevalence of PTSD and its associated risk factors (adjusted OR) for the CoV-2 pandemic are envisaged. Although strategies to resolve immediate PTSD are key, long-term PTSD must not be overlooked.

## 1. Introduction

Early in December 2019, a newly discovered infectious coronavirus (CoV-2) disease occurred in Wuhan, Hubei Province, China [1]. The appearance of coronavirus CoV-2 did not prove to be an easy matter. The rapid global spread of the disease led to the declaration of a pandemic on 11 March 2020 [1]. It was subsequently termed Coronavirus Disease 2019 (COVID-19) [2]. The primary reported manifestation of COVID-19 is severe acute respiratory distress syndrome (SARS), ultimately leading to death in the most severe cases [3]. It was named CoV-2 since CoV-1 appeared as a pandemic in 2003 and caused a similar effect of SARS [1]. CoV-2 and CoV-1 share similarities at several levels. Both viruses have a high degree of homology and they share similar clinical features and disease dynamics [4]. The disease progression follows a similar trend for CoV-2 and CoV-1, with SARS occurring approximately 8–20 days after the first symptoms [5]. While this has yet to be confirmed, the two outbreaks appear to have a different epidemic trajectory. Whereas the SARS outbreak was brought under control in a matter of 8 months in 2003, the new pandemic in 2019 continues after more than a year. Although shorter in duration, the CoV-1 pandemic caused a great deal of distress not only in patients but also in healthcare professionals (HCPs), being named a “mental health catastrophe” [6]. Although both CoV-1 and CoV-2 pandemics resulted from coronaviruses, their impacts on healthcare professionals were different. Several determinants of occupational and psychosocial distress have been reported in HCPs during and after the CoV-1 pandemic, suggesting the need to establish strategies to help physicians through measures including timely knowledge sharing, effective infection control practices, income protection during outbreaks, and attention to family risk management [7]. Given the considerably higher severity of the CoV-2 compared to the CoV-1 pandemic, lessons could be gained by evaluating the burdens of pandemics on HCP psychosocial distress, such as post-traumatic stress disorder (PTSD) [8]. Although the prevalence of PTSD in the CoV-2 pandemic can be predicted to be higher, common determinants of PTSD may be shared by both CoV-1 and CoV-2 pandemics.

The three hallmark features of a traumatic event as defined in DSM-5 (Diagnostic and Statistical Manual of Mental Disorders) are unpredictability, uncontrollability, and the threat of death or serious injury [9,10]. Pandemics, described as traumatic incidents, trigger a great deal of concern for HCPs and the health authorities. HCPs are passing through unprecedented challenges with the COVID-19 pandemic in many aspects. They are risking their own health and lives, threatened not only by exposure to the coronavirus but also by ever-increasing stress at the workplace, with amounts to a parallel the pandemic itself. Increased exposure to work-related stress has been associated with deleterious effects for mental health with higher rates of anxiety disorders. The recognition of early and long-term PTSD in healthcare professionals is becoming increasingly relevant for health policymakers to develop preventive measures to mitigate or avoid PTSD and related diseases. Dutheil et al. [1] described PTSD as the tsunami of the COVID-19 pandemic. PTSD is a severe mental health condition caused by an unusual traumatic life event beyond the normal range of human experience [11]. HCPs are under enormous pressure to manage this disaster, as they have to reorganize resources and the workforce to manage an unusual medical emergency. Being worried about their health, their families’ health, contagion, and their colleagues’ safety make them more prone to acute distress and potentially chronic PTSD [1,3]. Furthermore, the response by worldwide governments involving quarantine, social distance, lockdown measures, and media might contribute to PTSD [1,11,12]. We could, thus, overcome the pandemic, but then face a global public mental health crisis [13]. The pooled prevalence of PTSD symptoms among HCPs exposed to the COVID-19 pandemic ranged from 13% (95% confidence interval (CI): 11%, 16%) to 20.7% (95% CI: 13.2%, 31%) [14,15]. Previous systematic and meta-analysis reviews on the COVID-19 pandemic reported combined odds ratios (ORs) of risk factors for mental disorders (anxiety, depression, occupational stress, PTSD, and insomnia) among HCPs [16,17]. However, to our knowledge, no systematic reviews investigated risk factors associated with immediate and long-term PTSD related to the SARS (CoV-1 and CoV-2) pandemics.

In this systematic review, we aimed at investigating the scientific evidence on immediate and long-term PTSD effects on healthcare professionals due to the COVID-2019 (SARS CoV-2) and SARS-2003 (SARS CoV-1) pandemics. We systematically reviewed the literature for the prevalence of PTSD symptoms and associated risk factors (adjusted OR) due to exposure to coronavirus pandemics COVID-2019 (SARS CoV-2) and SARS-2003 (SARS CoV-1) among healthcare professionals (HCPs).

## 2. Methods

**Figure 1:**
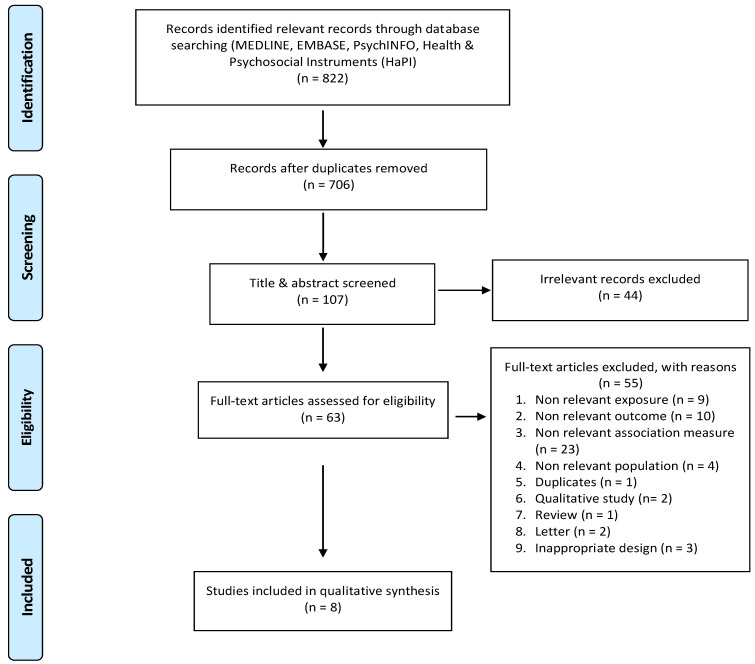
Flow diagram showing study selection for post-traumatic stress disorder (PTSD) related to coronavirus (CoV-1 and CoV-2) pandemics among healthcare professionals (HCPs).

This systematic review was conducted according to the recommendations of the Protocols for Systemic Review and Meta-Analysis (PRISMA) statement [18]. We included studies that focused on HCPs during pandemics COVID-2019 (SARS CoV-2) and SARS-2003 (SARS CoV-1). We narrowed our search to studies that focused on PTSD. The literature search was done on the following databases: Medline, Embase, PsychINFO, and Health Psychosocial Instrument (HaPI). The search was limited to primary studies published in peer-reviewed journals and in the English language from 1 January 2003 to 15 November 2020. As no cohort or case–control studies were found, only cross-sectional studies were included that reported prevalence of PTSD (frequency, %) and associated risk factors (adjusted OR) using multivariate regression analysis. Qualitative interview-based studies, review articles, editorials, opinion or letter articles, and studies on students or trainees were excluded. The studies retrieved from the search were imported into Covidence^®^. Covidence^®^ is an online systematic review software management tool which allows uploading search results, screening abstracts and full-text study reports, completing data collection, conducting risk of bias assessment, and resolving disagreements (Covidence systematic review software, Veritas Health Innovation, Melbourne, Australia. Available at www.covidence.org, accessed on 22 February 2021). Duplicated papers were removed. Three groups consisting of two residents (B.a.F. and M.a.D., M.a.A. and M.a.M., and A.a.A. and M.a.K.) shared the systematic reviewing process equally and independently screened the title and abstract of each article according to their relevance. A second screening process was undertaken which involved reviewing the full text for each article to assess its eligibility. Any disagreement between the reviewing groups was resolved by three senior supervisory members M.D., M.a.Q., and H.a.T. Figure 1 shows study selection process. Data extraction was done by three investigators using a structured form. This included study information (author name, year of population, country, and study design), population (study sample, total number of participants, age, data collection time period, and follow-up period), exposure (definition, measurement, and categorization by degree of exposure), outcome (psychosocial outcomes, definitions, ascertainment, and classification by severity), confounders, point of prevalence estimate for outcomes (frequency, %) exposure–outcome association measure (adjusted OR), and level of statistical significance (95% confidence intervals). As the studies included in this systematic review were all cross-sectional, the Joanna Briggs Institute Checklist for Analytical Approach for cross-sectional studies was used to assess their methodological quality [19].

## 3. Results

The electronic search identified 822 candidate studies, 706 of which remained after the elimination of duplicates (Figure 1). A total of 599 publications were excluded, and 107 remained for title and abstract screening, of which 44 were excluded and 63 remained for full-text examination, of which 55 were excluded, as the goal was to include cross-sectional and cohort studies that reported the prevalence of PTDS and carried out a regression analysis of possible risk factors. The final search resulted in a total of eight studies enrolled in the qualitative synthesis. The eight studies were of cross-sectional design and are summarized by characteristics in Table 1, quality in Table 2, and estimates (prevalence and odds ratio) in Table 3 [3,20,21,22,23,24,25,26]. Six of the eight studies reported PTSD symptoms among healthcare professionals during the COVID-19 pandemic in Asia (China [20,21,22], Singapore [3], and India [3]) and North America (United States of America [23,24]), while two studies were undertaken during the SARS-2003 pandemic in Asia (China [25] and Singapore [26]).

Sample size ranged from 263 to 5062 with a combined total of 10,074 participants. All of the studies self-reported level of exposure to coronaviruses (CoV-1 or CoV-2) and severity of PTSD symptoms using paper or online surveys with a response rate above 80% [3,25], of 60–80% [20,21,22,26], and below 15% [23,24]. Three studies were conducted in a single hospital [21,25,26], three studies were conducted in multiple hospitals [3,20,22], one study was conducted in a single state [23], and two studies were conducted in multiple states or provinces [20,24]. Two studies were investigated merely nursing staff [22,23], one study had a majority of female nursing staff (93.6%) [23], one study recruited just otolaryngology physicians [24], and two studies focused on nurses and doctors [20,26]. Three studies included allied HCPs and administrates [3,21,25]. All studies reported prevalence of acute or immediate PTSD symptoms during or immediately after the pandemic except one study [25] which reported delayed-onset or long-term PTSD (3 years after SARS-2003 pandemic). Evidently, to date, there is no study reporting long-term PTSD after CoV-2 pandemic.

All studies specified and categorized level of exposure with slight variations except one study [3] which did not report how the level of exposure was categorized. All studies used the Impact Event Scale (IES, 22-items, [3,20,21,22,25] and 15-items, [24,26]) with different cutoff scores to detect PTSD symptoms and their severity in the past 7 days except one study [25] which evaluated the past 4 weeks 3 years post SARS-2003 outbreak, whereas another study [23] used the six-item Post-Traumatic Checklist (PCL-6) in the past 4 weeks. All studies used logistic regression model to control and adjust for different risk factors and confounders.

### 3.1. Quality Assessment

Total quality scores for methodology were seven [20], six [3,21,23,24,25], and five [22] out of eight on the Joanna Briggs Institute Checklist for Analytical Approach for cross-sectional studies. None of studies used objective measurements or clinical interviews for PTSD symptoms (Table 2).

### 3.2. Prevalence and Determinants of Acute PTSD Symptoms

Regarding SARS CoV-1, the prevalence estimate of acute PTSD symptoms for Singapore was 3% [26].

Regarding SARS CoV-2, the prevalence of acute PTSD symptoms ranged between 25.1% and 71.5% for China [20,21,22] among HCPs, was 4% for Singapore [3], was 3.4% for India [3], and ranged between 26.5% and 60.2% for USA [23,24].

### 3.3. Determinants of Acute or Immediate PTSD

**Figure 2:**
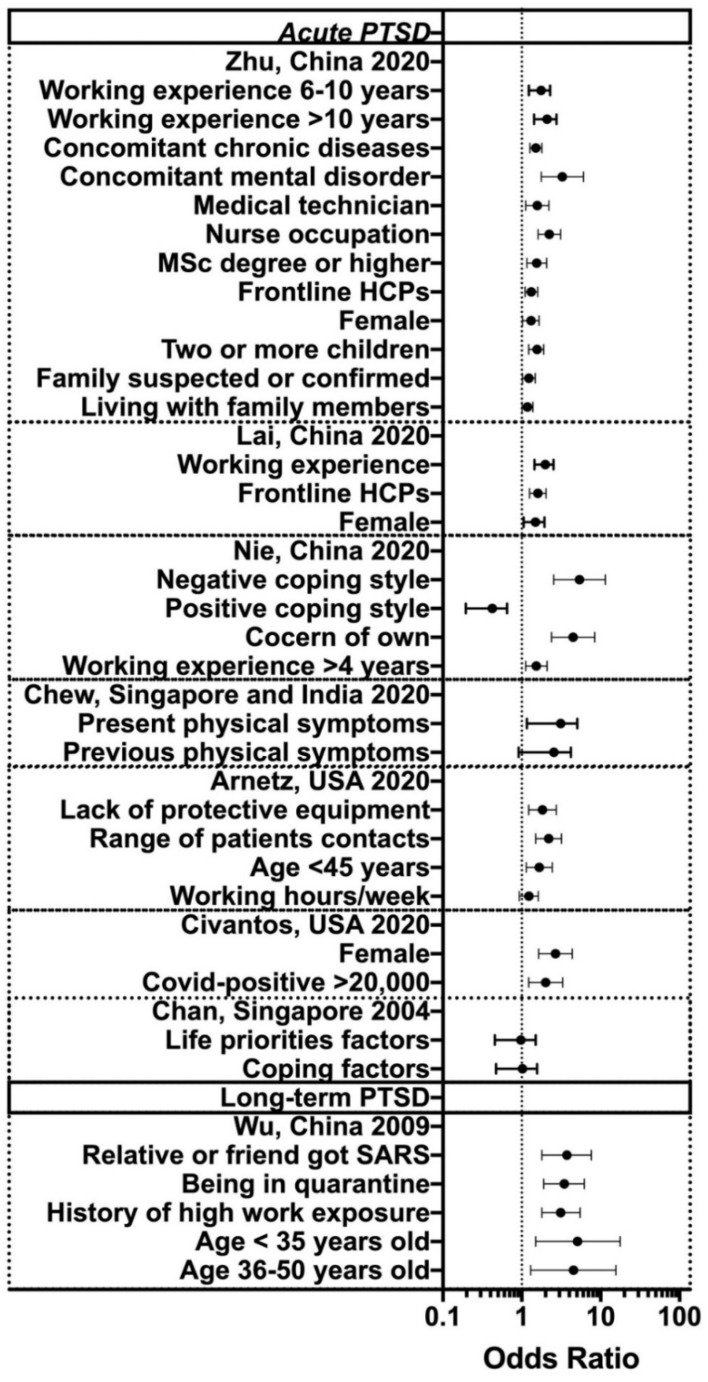
Determinants of acute and long-term PTSD.

The following variables have been shown by multivariable logistics regressive research to correlate with a higher risk of acute PTSD symptoms (Figure 2): age (<45 years old), OR 1.67 (95% CI: 1.14, 2.44) [23], female, OR between 1.31 (95% CI: 1.02, 1.66) [21] and 2.68 (1.64, 4.37) [24], working experience >4 years, OR between 1.53 (95% CI: 1.12, 2.10) [22] and >10 years, OR 2.02 (95% CI: 1.47, 2.79) [21], frontline HCPs engaged in direct diagnosis, treatment, and care of patients with COVID-19, OR 1.32 (95% CI: 1.10, 1.59) [21] to 1.60 (95% CI: 1.25, 2.04) [20], frequency of contact with COVID-19 patients, OR 2.19 (95% CI: 1.50, 3.19) [23], working hours/week, OR 1.23 (95% CI: 0.93, 1.62) [23], lacking access to adequate personal protective equipment (PPE), OR 1.83 (95% CI: 1.22, 2.74) [23], COVID-19 positive cases above 20,000 cases, OR 2.01 (95%CI: 1.22, 3.31) [24], and knowledge–attitudes–practices (KAP) factors such as concern of own, OR 4.48 (95% CI: 2.38, 8.42) and negative coping style, OR 5.40 (95% CI: 2.54, 11.46). Other risk factors reported by Zhu et al. [21] were education level of master’s degree or higher, OR 1.55 (95% CI: 1.16, 2.07), nurse occupation, OR 2.24 (95% CI: 1.61, 3.12), medical technician, OR 1.57 (95% CI: 1.12, 2.21), concomitant chronic diseases, OR 1.51 (95% CI: 1.27, 1.80) or mental disorder, OR 3.27 (95% CI: 1.77, 6.05), social status such as living with family members, OR 1.18 (95% CI: 1.01, 1.38), family members or relatives suspected or confirmed COVID-19 case, OR 1.23 (95%CI: 1.02, 1.48), and parental status of two or more children, OR 1.56 (95% CI: 1.22, 1.99). Chew et al. [3] found a bidirectional positive association between physical symptoms and PTSD, OR 2.70 (95% CI: 1.40, 5.24) and between PTSD and physical symptoms, OR 2.20 (95% CI: 1.12, 4.35).

Participants from outside Hubei province were associated with a lower risk of experiencing symptoms of PTSD compared with those in Wuhan, OR 0.62 (95% CI: 0.43, 0.88) [20]. Nie et al. [22] found that positive knowledge–attitudes–practices (KAP) and coping style could offer a protective element, OR 0.38 (95%CI: 0.22, 0.67).

### 3.4. Prevalence and Determinants of Long-Term PTSD Symptoms

Wu et al. [25] reported a prevalence of 10% for persistent PTSD symptoms among HCPs 3 years post SARS-2003 pandemic. They found that the following factors were associated significantly with higher risk of experiencing chronic PTSD symptoms (delayed and/or persistent 3 years after the pandemic was over): being in quarantine during the outbreak, OR 3.47 (95% CI: 1.9, 6.2), age below 35 years old, OR 5.08 (95% CI: 1.5, 17.7) or 36–50 years old, OR 4.54 (95% CI: 1.3, 15.6), history of high work exposure, OR 3.11 (95% CI: 1.8, 5.5), and relative or friend got SARS, OR 3.74 (95% CI: 1.8, 7.6).

## 4. Discussion

In this systematic review and meta-analysis, results were pooled from eight real-world observational studies that reported the prevalence of PTSD (frequency, %) and associated risk factors (adjusted OR) on healthcare professionals (HCPS) during pandemics SARS CoV-2 and SARS CoV-1. These studies mostly addressed clinically significant PTSD in the acute phase during and immediately following a pandemic, while only one study addressed long-term PTSD. As evidenced by the data collected in this review, PTSD symptoms showed high prevalence variations ranging from 3.4% [3] to 71.5% [20] among HCPs. Determinants of immediate PTSD were reported for the CoV-2 pandemic, while those for long-term PTDS were reported for the CoV-1 pandemic.

While very limited data on the course of clinically important PTSD were available, our findings can be considered broadly consistent with the results of two meaningful pandemic meta-analyses on HCPs’ mental health and pandemics [27,28]. While Pappa et al. studied the prevalence of depression, anxiety, and insomnia among HCPs during the COVID-19 outbreak [27], Allan et al. investigated the prevalence of common and stress-related mental health disorders in healthcare workers based in pandemic-affected hospitals [28]. Compared to Allan et al.’s meta-analysis [28], we limited our results to the SARS studies reporting odds ratios, while others did not [29,30,31,32,33,34] or had too small a sample size for regression analysis [35,36].

A series of early intervention trials aimed at individuals who were seen to be at high risk of PTSD development were prompted by a diagnosis of acute stress disorder [37]. Delayed-onset PTSD is defined as PTSD that develops at least 6 months after exposure to trauma, with cases of PTSD reportedly commencing years after the trauma occurrence [38]. Importantly, diagnosis during the acute phase after trauma is not intended to predict subsequent PTSD, but rather to describe people with elevated distress in the initial month who may benefit from mental health services [39]. No linear association could be identified between the severity of acute PTSD and the severity of delayed PTSD onset [40].

### 4.1. Prevalence of PTSD

In China, the highest prevalence reported was 71.5% [20] for acute PTSD symptoms among HCPs, while the lowest prevalence was 25.1% [22]. A possible explanation for the variation is the data collection period, as Lai et al. collected data from 29 January to 3 February 2020, i.e., during the initiation and acceleration phase of the COVID-19 outbreak curve and complete lockdown of Wuhan city, Hubei province, while Nie et al.’s data collection period was 3–10 February 2020 in Guangdong province, which was considered less affected by COVID-19 compared to Wuhan city, Hubei province [41,42,43,44].

In the USA, Civantos et al. [24] reported twofold higher prevalence of acute PTSD symptoms of 60.2%, compared to Arnetz et al. [23] (26.5%). One possible explanation for this discrepancy is the different use of self-reported tools used to ascertain PTSD. Civantos et al. [24] used the 15-item revised impact event scale (IES-R15) to detect symptoms over past 7 days among nurses (estimated response rate: 4%) during the month of May 2020, while Arnetz et al. [23] used the six-items Post-Traumatic Checklist (PCL-6) to detect PTSD symptoms in the past 4 weeks among otolaryngology physician (estimated repose rate: 10.2%) during the month of April.

The lowest prevalence of PTSD symptoms was reported in Singapore at 3% and 4% [3,26] and India at 3.4% [3]. There is a high variability in the association of traumatic events with PTSD, as it is not necessary for everyone exposed to a potentially traumatic event to develop a disorder [45].

Wu et al. [25] reported a prevalence of 10% for persistent PTSD symptoms among HCPs 3 years post SARS-2003 pandemic. Similar findings were reported by Maunder et al. [30], suggesting that impact of SARS can persist 1 to 2 years after the outbreak among HCPs compared with colleagues in settings that did not treat SARS patients.

### 4.2. Determinants of PTDS

Included studies in this review investigated varied determinants or risk factors associated with high prevalence of PTSD symptoms among HCPs. Gender, age, experience (working years), and degree of exposure (level, load, and amount of close contact or care) variables commonly showed significant association with high PTSD symptoms among different studies in different countries on different continents. As PTSD determinants, personal characteristics, conditions, and energies are resources of both instrumental and symbolic usefulness. Personal characteristics such as a positive attitude, view of events as predictable, and the ability to deal with stress are ways to help protect against stress [46]. Personal conditions, such as job training and seniority, are resources that are valued and sought after by an individual [47]. Any provided condition may be a resource for one person but may be harmful for another [48]. Personal energy resources include time, money, and expertise. This type of resource is useful, since it can be used to obtain additional resources [48,49].

## 5. Strengths and Limitations of the Study

This review had several strengths. It included research on two pandemics in Asia and North America, including investigation of PTSD symptoms with further regression analyses of possible risk factors. It demonstrated and highlighted the immediate and delayed traumatic effects of two pandemics in various settings and subpopulations of HCPs. This systematic review, therefore, provides valuable information and may serve as a guide for policymakers to establish and provide guidance on immediate and long-term traumatic stress among HCPs.

This review had several limitations. The search was limited to English language only and only those published in peer-reviewed journals; thus, it could be prone to publication bias. The studies were all cross-sectional studies, with a short follow-up period; therefore, the studies could not prove causality. Since all studies were self-reported with different cutoff scales and different versions of the Impact Event Scale (IES), and since no study utilized standardized clinical interviews for diagnosis as a confirmation tool, it is unknown whether the presence of an actual disorder existed, which could have led to misclassification and recall biases. However, in such pandemic situations, this was not a feasible way to conduct studies as direct contact was restricted. Nevertheless, it could be possible to investigate delayed or persistent PTSD once the pandemic is over. Furthermore, the majority of studies were heterogeneous in their operational assessment of exposure and used population screening scales to assess PTSD determinants with different cutoff scores. Therefore, summarizing the prevalence of PTSD and its determinants among HCPs with a single-point estimate was difficult, and reported findings need to be interpreted with cautions.

When used alone, the OR, which remains a representation of the power of association between the risk factor and the onset of PTSD, offers little detail. Meta-analyses represent an accumulation of knowledge that can often lead to meanings that do not address the clinical validity issue and leave the decision to be made in confusion [50]. This approach must then be used with experience and moderation and, where possible, should be supplemented by additional research illustrating the clinical validity of the meanings acquired.

## 6. Conclusions

A comprehensive understanding of the prevalence and determinants of immediate or long-term PTSD related to pandemics for healthcare workers may enhance its prevention, diagnosis, and management. Working experience, occupation, protective conditions, concomitant chronic diseases, present physical symptoms, mental disorder, negative coping style, and family-related factors were identified as determinants of an immediate PTSD. Exposure to SARS, being in quarantine, high work exposure, and age were reported as determinants of a long-term PTSD. The risk of immediate or long-term PTSD can be decreased on the basis of identifiable risk factors.

Studies are yet to investigate the long-term consequences of PTSD after a CoV-2 pandemic over longer periods of time. Rigorous studies evaluating the prevalence of PTSD and its related risk factors (adjusted OR) for the CoV-2 pandemic are envisaged. While strategies to tackle immediate PTSD are key, strategies for long-term PTSD must not be ignored.

## Figures and Tables

**Table 1:** Characteristics of COVID-2019 disease (severe acute respiratory distress syndrome (SARS) CoV-2) studies (population, exposure, outcomes, and confounders). USA, United States of America; PPE, personal protective equipment.

Author, Year, Country, Study Design	Population Study Sample, Total Number of Participants (Response Rate%), Sociodemographic Data, Data Collection Period	Exposure Traumatic Event: Coronavirus (CoV-2) Pandemic Definition, Measurement, Categorization	Outcome Post-Traumatic Stress Disorder (PTSD) Definition, Ascertainment Classification	Confounders
**COVID-2019 Disease (SARS CoV-2)**				
Zhu [21], 2020, China, Cross-sectional	6568 healthcare workers (HCW) in a single center, Tongji Hospital (Wuhan) were surveyed. 5281 individuals completed the online questionnaire. 5062 HCWs (all doctors, nurses, and clinical technicians) were included in the final analysis (response rate, 77.1%). Administrates and incomplete questionnaires were excluded. The response rates of female and male HCWs were 82.4% and 71.8%. Most subjects were in the age intervals of 19–29 (40.1%) and 30–49 years old (56.4%). Data collection period: 8–10 February 2020, 2 weeks after transport suspension	**Definition:** frontline HCWs directly in contact with confirmed or suspected Covid-19 cases. **Measurement:** self-reported frontline HCWs working in isolation ward, fever clinic, or emergency department. **Categorization:** level of exposure classified by current workplace (isolation ward, non-isolation ward, off work or in isolation), department (fever clinic, emergency department or isolation ward, non-isolation ward, another department), present at frontline (yes or no), COVID-19 infection status of families or relatives or self-suspected or confirmed (yes or no)	**Definition:** acute psychological stress **Ascertainment:** self-reported 22-item revised Impact Event Scale (IES-R) to assess three subjective acute stress symptoms (avoidance, intrusion, and hyperarousal) caused by traumatic event. The IES-R was validated in previous COVID-19 studies which provided adequate specificity (91.0%) and sensitivity (82.0%). **Classification:** IES-R scores (<33 or >33); scores >33 points were used to identify outcome of stress.	Age, gender, marital and social status, education level, occupation, years of experience, annual income, past medical history, smoking, drinking, physical activity level
Lai [20], 2020, China, Cross-sectional	Healthcare workers (HCW) in 34 hospitals stratified by provinces and regions. Hospitals outside Hubei province: 7 hospitals, 1 COVID-19 hospital in each province Regional hospitals inside Hubei province: 27 hospitals 20 hospitals in Wuhan region: 10 COVID-19 and 10 non COVID-19 Other regions: 7 COVID-19 hospitals, 1 COVID-19 hospital in each region One clinical department was randomly sampled from each selected hospital, and all HCWs in this department were asked to participate in this study. HCWs included physicians and nurses. The target sample size of participants was calculated and amplified: 1070 participants were required. 1257 of 1830 contacted individuals completed survey (response rate: 68.7%). Of the 1257 responding participants, 493 (39.2%) were physicians, and 764 (60.8%) were nurses. 760 (60.5%) worked in Wuhan, 261 (20.8%) worked in Hubei province outside Wuhan, and 236 (18.8%) worked outside Hubei province. Most participants were women (964, 76.7%), aged 26 to 40 years (813, 64.7%), married, widowed, or divorced (839, 66.7%), had an educational level of undergraduate or less (953, 75.8%), had a junior technical title (699, 55.6%), and worked in tertiary hospitals (933, 74.2%). A total of 522 participants (41.5%) were frontline HCWs. Nearly all participants (1220, 97.1%) lived in urban areas. Data collection period: 29 January–3 February 2020	**Definition:** HCW in hospitals equipped with fever clinics or wards for patients with COVID-19. **Measurement:** self-reported exposure to COVID-19 patients in fever clinics or wards during outbreak. **Categorization:** level of exposure stratified by Geographical location: Wuhan, Hubei province, outside Wuhan, or outside Hubei province Type of hospital: secondary or tertiary Working position: frontline (directly engaged in clinical activities of diagnosing, treating, or providing nursing care to patients with elevated temperature or patients with confirmed COVID-19). If answered no, then defined as second-line	**Definition:** symptoms of distress **Ascertainment:** self-reported 22-item Impact of Event IES-R. **Classification:** severity of symptoms classified as normal (0–8), mild (9–25), moderate (26–43), and severe (44–88) distress. Cutoff score > 26 for defined as severe distress.	Gender, age, marital status, educational level, technical title, place of residence, working position (first-line or second-line), and type of hospital
Nie [22], 2020, China, Cross-sectional	263 nurses of the frontline department from seven designated hospitals for COVID-19 patients in Guangdong Province participated in the survey (response rate 30–40%). Of the 263 participants, 202 (76.7%) were female and the majority (*n* = 236, 89.7%) were younger than 39 years of age. The most common (*n* = 189, 71.9%) educational qualification was undergraduate or above and the majority (*n* = 196, 74.5%) worked in the emergency department. Data collection period: 3–11 February 2020	**Definition:** frontline nurses directly in contact with infected or suspected COVID-19 patients. **Measurement:** self-reported working on the frontline department such as emergency department, fever clinic, isolation ward, intensive care unit (ICU), and infection department. **Categorization:** level of exposure classified emergency department versus non-emergency department	**Definition:** symptoms of post-traumatic stress disorder (PTSD). **Ascertainment:** self-reported 22-item revised Chinese version of the Impact of Event Scale (IES-R) was used to evaluate intrusive thoughts related to COVID-19 and consequent avoidance behavior. It was divided into three dimensions: intrusion, avoidance, and hyperarousal. **Classification:** 4-point Likert scale (scores 0–4) was adopted to assess the IES in the past 7 days. Participants with a score greater than or equal to 20 were interpreted to be affected by traumatic event.	Gender, age, educational level, marital status, working department, working years.
Chew [3], 2020, Singapore and India, Cross-sectional	Healthcare workers (HCWs) from 5 COVID-19 major tertiary hospitals in Singapore and India. HCWs included doctors, nurses, allied HCWs (pharmacists, physiotherapists, occupational therapists, technicians), administrators, clerical staff, and maintenance workers. Of the 1000 invited HCPs, 906 agreed to participate (response rate: 90.6%): 480 respondents from Singapore and 426 from India Majority (583, 64.3%) were female and the median age was 29; most (55.1%) of the participants were Indian, followed by Chinese (33.7%) and Malay (4.8%) ethnicity. 50.2% of the participants were unmarried. 205 (22.6%) participants had pre-existing comorbidities, with migraine (9.6%) being the most prevalent followed by eczema (4.1%) and asthma (4.0%). Nurses comprised 39.2% of the study population, followed by physicians (29.6%) and allied healthcare professionals (10.6%) Data collection period: 19 February–17 April 2020	**Definition:** HCWs exposed to COVID-19 patients. **Measurement:** self-reported exposure to care of COVID-19 patients in major tertiary healthcare institutions during the outbreak. **Categorization:** level of exposure not specified	**Definition:** psychological distress/impact during coronavirus disease 2019 (COVID-19) outbreak. **Ascertainment:** self-reported 22-item Impact of Events Scale Revised (IES-R25), divided in three subscales (intrusion, avoidance, hyperarousal) validated to measure subjective distress symptoms during past 7 days caused by traumatic event in Chinese general population during COVID-19 **Classification:** degree of severity graded by IES-R25 score as normal (0–23), mild (24–32), moderate (33–36), or severe (>37). A cutoff score of 24 used to define PTSD of clinical concern.	Age, gender, presence of comorbidities
Arnetz [23], 2020, USA, Cross-sectional	Participants were recruited from the Michigan chapter of the American Nurses Association (ANA), the Michigan Organization of Nurse Leaders (MONL), and the Coalition of Michigan Organizations of Nursing (COMON). All members of the three organizations (~18,300) and their colleagues were eligible. Calculated sample size (*n* = 580). A total of 695 nurses responded to the survey (response rate estimated at 4%) Most of the respondents were female (*n* = 644, 93.6%), older than 45 (*n* = 376, 54.7%), Caucasian (*n* = 611, 87.9%), and had been working for more than 10 years (*n* = 449, 67.1%). Majority (*n* = 533, 90.0%) worked in urban locations, more than half worked 20–40 h per week (*n* = 368, 56.6%), and 36.6% (*n* = 238) worked 41–60 h per week or more. Nearly 60% (*n* = 392, 59.1%) worked in an inpatient setting and 19.7% (*n* = 135) held a management position. Forty percent (*n* = 269; 40.2%) reported being in frequent contact with COVID-19 patients while 24.9% (*n* = 163) reported not being provided with adequate PPE by their workplace. Data collection period: May 2020	**Definition:** exposure to COVID-19 patients and access to personal protective equipment (PPE). **Measurement:** level of exposure by answering single-items about frequency of contact with COVID-19 patients, access to adequate PPE and number of hours worked per week, practice setting, managerial position. **Categorization:** Frequency of contact with COVID-19 patients: 4-point response scale from never to very often. Access to adequate PPE: 4-point scale from not at all to definitely; not applicable could also be selected. Number of hours worked per week: <20, 20–40, 41–60, >60. Practice setting: inpatient versus outpatient. Management position: yes or no	**Definition:** symptoms of post-traumatic stress disorder (PTSD). **Ascertainment:** self-reported 6-item Post-traumatic Checklist (PCL-6). An abbreviated version of 20-item PTSD Checklist screening for PTSD symptoms of “repeated, disturbing memories, thoughts, or images of a stressful experience from the past”, “feeling very upset when something reminded you of a stressful experience from the past”, avoided activities or situations because they reminded you of a stressful experience from the past, feeling distant or cut off from other people, feeling irritable or having angry outbursts, and difficulty concentrating for the past 4 weeks. **Classification:** severity of PTSD symptoms rated on a scale from 1 (not at all) to 5 (extremely). The cutoff score for presence of PTSD symptoms was 14.	Age, gender, race, number of hours worked per week, years, working as a nurse, working in a management position, geographic location, and work practice setting (inpatient versus outpatient/ community)
Civantos [24], 2020, USA, Cross-sectional	A total of 349 otolaryngology physicians (residents, fellows, attendings, and physicians) at the academic institutions across the United States participated in the national survey. A total of 1614 otolaryngology residents and 2849 otolaryngology fellows and attendings work was estimated (response rate was 10.22%). Of these, 165 (47.3%) were residents, and 184 (52.7%) were attending physicians, of which 12 were fellows. Most participants were men (212, 60.7%), and the most common age range was 31–35 years (114, 32.7%). A number of 126 (36.1%) participants worked in the Midwest, 107 (30.7%) worked in the Northeast, 75 (21.5%) worked in the South, and 41 (11.7%) worked in the West. The majority came from states projected to reach their peak resource use during the study period (205, 58.7%). Accordingly, 54.2% of participants came from states estimated to have greater than 20 000 confirmed positive COVID-19 cases, and 54.2% came from states estimated to have greater than 1000 COVID-19 deaths. Data collection period: 14 April 2020 to 25 April 2020.	**Definition:** peak of resource utilization for each state during COVID-19 outbreak. **Measurement:** Date of projected peak resource utilization Institute for Health Metrics and Evaluation’s COVID-19 Projections. **Categorization:** level of participants’ exposure was categorized by state’s surge status into pre-surge, surge, and post-surge on the basis of number of positive COVID-19 cases (< or >20,000) and COVID-19 deaths (< or >1000) published on Institute for Health Metrics and Evaluation’s Covid-19 Projections.	**Definition:** psychological distress (PTSD) symptoms **Ascertainment:** self-reported 15-item Impact of Event Scale (IES, score range: 0–75) to assess symptoms of PTSD over the past 7 days. The IES total score was also divided into two sub scores: intrusion (range: 0–35) and avoidance (range: 0–40). **Classification:** severity of PTSD symptoms was classified as subclinical (0–8), mild (9–25), moderate (26–43), and severe (44–75) distress. A score of 27 was reported as a cutoff for risk of post-traumatic stress disorder (PTSD).	Type of physician, sex, age, surge status, and number of positive cases
**SARS-2003 (SARS CoV-1)**				
Wu [25], 2009, China, Cross-sectional	Healthcare workers (HCWs) from a major hospital in Beijing that had been affected by the 2003 SARS outbreak 549 HCWs participated and were stratified by 3 professional categories: doctor, nurse, and administrative and/or other hospital staff (response rate 83%). Three-fourths of the sample were women; 47% were aged between 36 and 50 years; 19% were aged 50 years or older Data collection period (2006, 3 years post SARS-2003 outbreak)	**Definitions:** exposure to SARS outbreak as a traumatic event related to work, any quarantining, having a friend or close relative who contracted SARS, media, and other traumatic events. **Measurement:** self-reported profession (doctor, nurse, technician, others), work exposure (working in a high-risk location, such as a SARS ward, fever clinic, infectious disease department, emergency room, pulmonary medicine department, or X-ray laboratory), quarantine (as a result of being diagnosed with SARS or suspected of having SARS, or as having had direct contact with SARS patients either at work, at home, or in other places), relative or friend got SARS (having one or more family members or friends who developed SARS, and either died from or recovered from it), media (amounts of exposure to coverage about the SARS outbreak the hospital employees had received, through 3 types of media: television, websites, and other (radio, newspapers, or magazines)), and other traumatic events (any potentially traumatic event prior to and following the SARS-2003 outbreak (severe injury in violent circumstances, witnessing a death or serious injury of a close friend or family member, and living through a major disaster). **Categorization:** level of exposure for doctor and nurse was classified as either high (who worked in units such as SARS wards, fever clinics, the department of infectious diseases, or the emergency room, where contact with SARS patients was frequent and intense) or low.	**Definition:** persistence of post-traumatic stress disorder (PTSD) symptoms 3 years post Beijing’s SARS-2003 outbreak **Ascertainment:** self-reported 22-item Impact of Events Scale Revised (IES-R) that was translated and validated in Chinese to assess subjective distress symptoms resulting from a traumatic life event persisting over the past month. **Classification:** Likert rating scale from 0 to 4; the total score had a range of 0 to 88. Score of 20 or more indicated high level of PTSD symptoms	Sociodemographic variables (age, gender, family income, educational level), prior exposure to trauma, perceived risk during the SARS outbreak, altruistic acceptance of risk
Chan [26], 2004, Singapore, Cross-sectional	Medium-size regional general hospital. 993 total nurses and doctors; exposed 147, unexposed 846 661 responded: 113 doctors, 544 nurses (response rate: 67%) Data collection period (May 2003, 2 months post outbreak)	**Definition:** exposure or SARS in a regional hospital 2 months after the first case of SARS was reported. **Measurement:** self-reported exposure of being contact with suspect or probable SARS patients (yes, no or not sure), workplace (intensive care unit, emergency department, fever ward, general, others) **Categorization:** sample classified into 2 groups on the basis of level of exposure Group A: HCWs who were first-generation contacts or who had direct contact with suspect or probable SARS patients (total: 106, doctors: 32, nurses: 74) Group B: HCWs who did not have direct contact with any suspect or probable SARS patients (total: 555, doctors: 81 nurses: 474)	**Definition:** Post-traumatic stress disorder (PTSD) symptoms among HCWs exposed to SARS outbreak. **Ascertainment:** self-reported 15-item Impact of Events Scale (IES-15) to assess PTSD symptoms. **Classification:** PTSD present or absent; IES score > 30 was chosen for indicating presence.	Age, race, marital status, and workplace

**Table 2:** Quality assessment of included studies.

Author, Year	1	2	3	4	5	6	7	8	Total (/8)
**COVID-2019 disease (SARS CoV-2)**									
Zhu [21]	Y	Y	Y	N	Y	Y	N	Y	6
Lai [20]	Y	Y	Y	N	Y	Y	Y	Y	7
Nie [22]	Y	Y	N	N	Y	Y	N	Y	5
Chew [3]	Y	Y	N	N	Y	Y	Y	Y	6
Arnetz [23]	Y	Y	Y	N	Y	Y	N	Y	6
Civantos [24]	Y	Y	N	N	Y	Y	Y	Y	6
**SARS-2003 (SARS CoV-1)**									
Wu [25]	Y	Y	Y	N	Y	Y	N	Y	6
Chan [26]	Y	Y	Y	N	Y	Y	N	Y	6

1. Were the criteria for inclusion in the sample clearly defined? 2. Were the study subjects and the setting described in detail? 3. Was the exposure measured in a valid and reliable way? 4. Were objective, standard criteria used for measurement of the condition? 5. Were confounding factors identified? 6. Were strategies to deal with confounding factors stated? 7. Were the outcomes measured in a valid and reliable way? 8. Was appropriate statistical analysis used? Y: yes, N: no, NA: not available.

**Table 3:** Summary of studies that reported prevalence (frequency, %), severity, and factors significantly associated with post-traumatic stress disorder (PTSD) symptoms following multivariable logistic regression for COVID-19 disease (SARS CoV-2) and SARS-2003 (SARS CoV-1).

Publication (Author, Year, Country)	Outcome PTSD Symptoms (Frequency, %) Severity Categories of Traumatic Stress Symptoms	Association Measure: Odds Ratio (OR), 95% Confidence Interval (CI) Factors Significantly Associated with PTSD
**COVID-2019 Disease (SARS CoV-2)**		
Zhu [21], 2020, China	Study sample size *n* = 5062 Overall prevalence of PTSD symptoms IES-R22 cutoff score > 33 for detecting symptoms, past 7 days 1506/5062 (29.8%) Severity categories of traumatic stress symptoms Not reported	Gender Female: 1.31 (1.02, 1.66) Education level Master’s degree or higher: 1.55 (1.16, 2.07) Occupation Nurse: 2.24 (1.61, 3.12) Medical technician: 1.57 (1.12, 2.21) Years of working 6–10 years: 1.71 (1.25, 2.30) >10 years: 2.02 (1.47, 2.79) Department/working position Isolation ward: 1.32 (1.10, 1.59) Past medical history Positive for chronic disease: 1.51 (1.27, 1.80) Positive for mental disorder: 3.27 (1.77, 6.05) Social status Living with family members: 1.18 (1.01, 1.38) Family members or relatives suspected or confirmed COVID-19 case: 1.23 (1.02, 1.48) Parenteral status Two or more children: 1.56 (1.22, 1.99)
Lai [20], 2020, China	Study sample size *n* = 1257 Overall prevalence of PTSD symptoms IES-R22 cutoff score > 26 for detecting “severe” symptoms, past 7 days 899/1257 (71.5%) Severity categories of traumatic stress symptoms Normal (0–8): 358/1257 (28.5%) Mild (9–25): 459/1257 (36.5%) Moderate (26–43): 308/1257 (24.5%) Severe (44–88): 132/1257 (10.5%)	Gender Female: 1.45 (1.08–1.96) Working years/Technical title Intermediate: 1.94 (1.48, 2.55) Department/working position Frontline: 1.60 (1.25, 2.04) Geographical location Outside Hubei province: 0.62 (0.43, 0.88)
Nie [22], 2020, China	Study sample size *n* = 263 Overall prevalence of PTSD symptoms IES-R22 cutoff score >20 for detecting symptoms, past 7 days 66/263 (25.1%) Severity categories of traumatic stress symptoms Not reported	Working years (>4 years) 1.53 (1.12, 2.10) Concern of own 4.48 (2.38, 8.42) Negative coping style 5.40 (2.54, 11.46) Positive coping style 0.38 (0.22, 0.67)
Chew [3], 2020, Singapore and India	Study sample size *n* = 906 Overall prevalence of PTSD symptoms IES-R22 cutoff score >24 for detecting symptoms, past 7 days 67/906 (7.4%) Singapore (*n* = 480) 36/906 (4%) India (*n* = 426) 31/906 (3.4%) Severity categories of traumatic stress symptoms Normal (0–23): 11 (3.6%) Mild (24–32): 33 (3.6%) Moderate (33–36): 14 (1.5%) Severe (> 37): 20 (2.2%)	Present physical symptoms 2.70 (1.40–5.24) Previous physical symptoms 2.20 (1.12–4.35)
Arnetz [23], 2020, USA	Study sample size *n* = 695 Overall prevalence of PTSD symptoms PCL-6 cutoff score >14 for detecting symptoms, past 4 weeks 184/695 (26.5%) Severity categories of traumatic stress symptoms Not reported	Age <45 years: 1.67 (1.14, 2.44) Number of hours worked/week 1.23 (0.93, 1.62) Contact with COVID-19 patients Often/ very often: 2.19 (1.50, 3.19) Workplace provided adequate PPE No/not really: 1.83 (1.22, 2.74)
Civantos [24], 2020, USA	Study sample size *n* = 349 Overall prevalence of PTSD symptoms IES-R15 cutoff score >27 for detecting symptoms, past 7 days 210/349 = 60.2% Severity categories of traumatic stress symptoms Subclinical (0–8): 139/349 (39.8%) Mild (9–25): 114/349 (32.7%) Moderate (26–43): 73/349 (20.9%) Severe (44–75): 23/349 (6.6%)	Gender Female: 2.68 (1.64, 4.37) Covid-19 positive cases >20,000 cases: 2.01 (1.22, 3.31)
**SARS-2003 (SARS CoV-1)**		
Chan [26], 2004, Singapore	Study sample size *n* = 661 Overall prevalence of PTSD symptoms IES-R15 cutoff score > 30 for detecting presence of PTSD, past 7 days 20/661 (3%) Doctors (*n* = 6) 6/906 (0.9%) Nurses (*n* = 14) 14/906 (2.1%) Severity categories of traumatic stress symptoms Not reported	Life priorities factors 0.88 (0.51, 1.54), statistically not significant Coping factors 0.92 (0.53, 1.61), statistically not significant
Wu [25], 2009, China (past 3 years)	Study sample size *n* = 549 Overall prevalence of delayed or persistent PTSD symptoms (3 years post SARS) IES-R22 cutoff score > 20 for detecting high level symptoms, past 4 weeks 55/549 = 10% Severity categories of traumatic stress symptoms Not reported	Age <35 years: 5.08 (1.5–17.7) 36–50 years: 4.54 (1.3–15.6) High work exposure 3.11 (1.8–5.5) Any quarantine 3.47 (1.9–6.2) Relative or friend got SARS 3.74 (1.8–7.6)

PCL-6: 6-item post-traumatic checklist; IES: impact of event scale.

## Data Availability

Data sharing not applicable. No new data were created or analyzed in this study. Data sharing is not applicable to this article.
